# Crystal structures of {μ_2_-*N*,*N*′-bis­[(pyridin-3-yl)meth­yl]ethanedi­amide}tetra­kis­(di­methyl­carbamodi­thio­ato)dizinc(II) di­methyl­formamide disolvate and {μ_2_-*N*,*N*′-bis­[(pyridin-3-yl)meth­yl]ethanedi­amide}tetra­kis­(di-*n*-propyl­carbamodi­thio­ato)dizinc(II)

**DOI:** 10.1107/S2056989017012956

**Published:** 2017-09-19

**Authors:** Hadi D. Arman, Pavel Poplaukhin, Edward R. T. Tiekink

**Affiliations:** a Department of Chemistry, The University of Texas at San Antonio, One UTSA Circle, San Antonio, Texas 78249-0698, USA; b Chemical Abstracts Service, 2540 Olentangy River Rd, Columbus, Ohio, 43202, USA; c Research Centre for Crystalline Materials, School of Science and Technology, Sunway University, 47500 Bandar Sunway, Selangor Darul Ehsan, Malaysia

**Keywords:** crystal structure, zinc, di­thio­carbamate, di-amide, hydrogen bonding

## Abstract

The title structures, [Zn_2_(C_3_H_6_NS_2_)_4_(C_14_H_14_N_4_O_2_)]·2C_3_H_7_NO (I) and [Zn_2_(C_7_H_14_NS_2_)_4_(C_14_H_14_N_4_O_2_)] (II), each feature a bidentate, bridging bipyridyl-type ligand encompassing a di-amide group. In (I), the binuclear compound is disposed about a centre of inversion, leading to an open conformation, while in (II), the complete mol­ecule is completed by the application of a twofold axis of symmetry so that the bridging ligand has a U-shape. In each of (I) and (II), the di­thio­carbamate ligands are chelating with varying degrees of symmetry, so the zinc atom is within an NS_4_ set approximating a square-pyramid for (I) and a trigonal-bipyramid for (II). The solvent di­methyl­formaide (DMF) mol­ecules in (I) connect to the bridging ligand *via* amide-N—H⋯O(DMF) and various amide-, DMF-C—H⋯O(amide, DMF) inter­actions. The resultant three-mol­ecule aggregates assemble into a three-dimensional architecture *via* C—H⋯π(pyridyl, chelate ring) inter­actions. In (II), undulating tapes sustained by amide-N—H⋯O(amide) hydrogen bonding lead to linear supra­molecular chains with alternating mol­ecules lying to either side of the tape; no further directional inter­actions are noted in the crystal.

## Abstract 2

The Zn^II^ atom is each of the title compounds is coordinated by two di­thio­carbamate ligands and a pyridyl-N atom. The resultant NS_4_ donor set approximates a square-pyramid and trigonal-bipyramid, for the solvated and unsolvated structures, respectively. In the solvate, amide-N—H⋯O(di­methyl­formamide) hydrogen-bonds define a three-mol­ecule aggregate while in the unsolvated structure, amide⋯amide hydrogen-bonding leads to a supra­molecular chain.

## Chemical context   

The potential of self-association between amide functionalities *via* amide-N—H⋯O(amide) hydrogen-bonding has long been recognized (MacDonald & Whitesides, 1994 ▸). In this way, eight-membered {⋯HNCO}_2_ synthons can be formed. Alternatively, extended aggregation patterns based on a single point of contact repeat associations leading to supra­molecular chains or double-connections (edge-shared) leading to tapes. In this connection, isomeric di-amide structures of the general formula (*n*-NC_5_H_4_)CH_2_N(H)C(=O)—C(=O)N(H)CH_2_(C_5_H_4_N-*n*), for *n* = 2, 3 and 4, hereafter abbreviated as *^n^L*H_2_, have long attracted inter­est for their potential to form supra­molecular tapes. For example, as realized in the two-dimensional structure formed in the 1:1 co-crystal of ^4^ *L*H_2_ and the conformer, bi-functional 1,4-di-iodo­buta-1,3-diyne (Goroff *et al.*, 2005 ▸). Here, the amide tapes are orthogonal to the N⋯I halogen bonding. In the realm of metal-containing species, a three-dimensional architecture can be assembled in the crystal of {[Ag(^3^ *L*H_2_)_2_]BF_4_}_*n*_ by a combination of Ag←N bonds for the tetra­hedral silver(I) atom, provided by bidentate bridging ligands, where the latter are also connected *via* concatenated {⋯HNC_2_O}_2_ synthons (Schauer *et al.*, 1997 ▸).
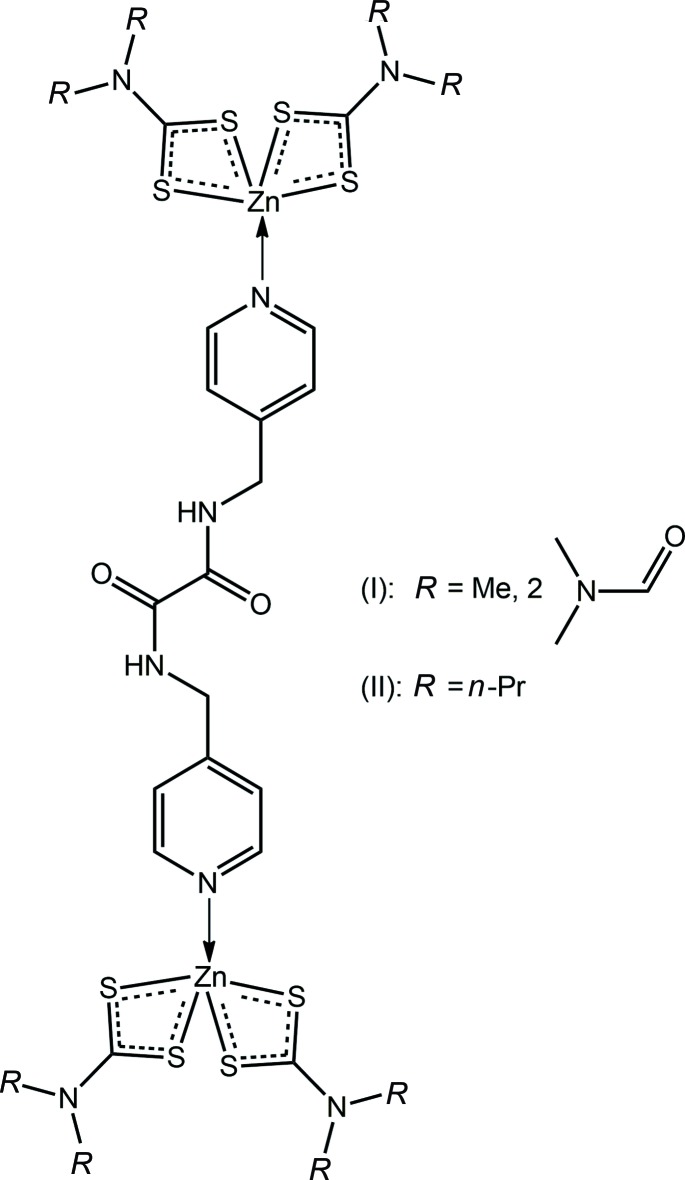


A similar coordination/hydrogen-bonding arrangement is found in the three-dimensional assembly in crystals of {[Cu(^3^ *L*H_2_)_2_Br]·Br·H_2_O}_*n*_ (Zeng *et al.*, 2008 ▸). Motivated by these results, investigations were commenced exploring the coordination ability of *^n^L*H_2_ with zinc(II) di­thio­carbamates functionalized with hydrogen-bonding potential, *i.e*. Zn[S_2_CN(*R*)CH_2_CH_2_OH]_2_, for *R* = alkyl, CH_2_CH_2_OH. As discussed in more detail in the *Database survey*, none of these crystals exhibited self-association of the amide residues. For example, in the crystal of binuclear {Zn[S_2_CN(Me)CH_2_CH_2_OH]_2_}_2_(^3^ *L*H_2_), supra­molecular chains are constructed as a result of hy­droxy-O—H⋯O(hy­droxy) hydrogen bonding that leads to the formation of sterically unencumbered 28-membered {⋯HOC_2_NCSZnSCNC_2_O}_2_ synthons. Two chains inter-weave through these rings and are held in place by hy­droxy-O—H⋯O(amide) hydrogen bonding (Poplaukhin & Tiekink, 2010 ▸). In a continuation of these studies, attention was directed towards the inter­action of *^n^L*H_2_ with all-alkyl zinc(II) di­thio­carbamates with the view of ‘turning-off’ putative hy­droxy-O—H⋯O(amide) hydrogen bonding. Herein, the crystal and mol­ecular structures of [Zn(S_2_CNMe_2_)]_2_(^3^ *L*H_2_)·2DMF [(I); DMF = di­methyl­formamide] and {Zn[S_2_CN(*n*-Pr)_2_]_2_}_2_(^3^ *L*H_2_), (II), are described where amide-N—H⋯O(DMF) hydrogen bonding precludes supra­molecular association *via* amide⋯amide hydrogen bonding in (I), but not in (II), where supra­molecular amide tapes are observed.

## Structural commentary   

**Figure 1:**
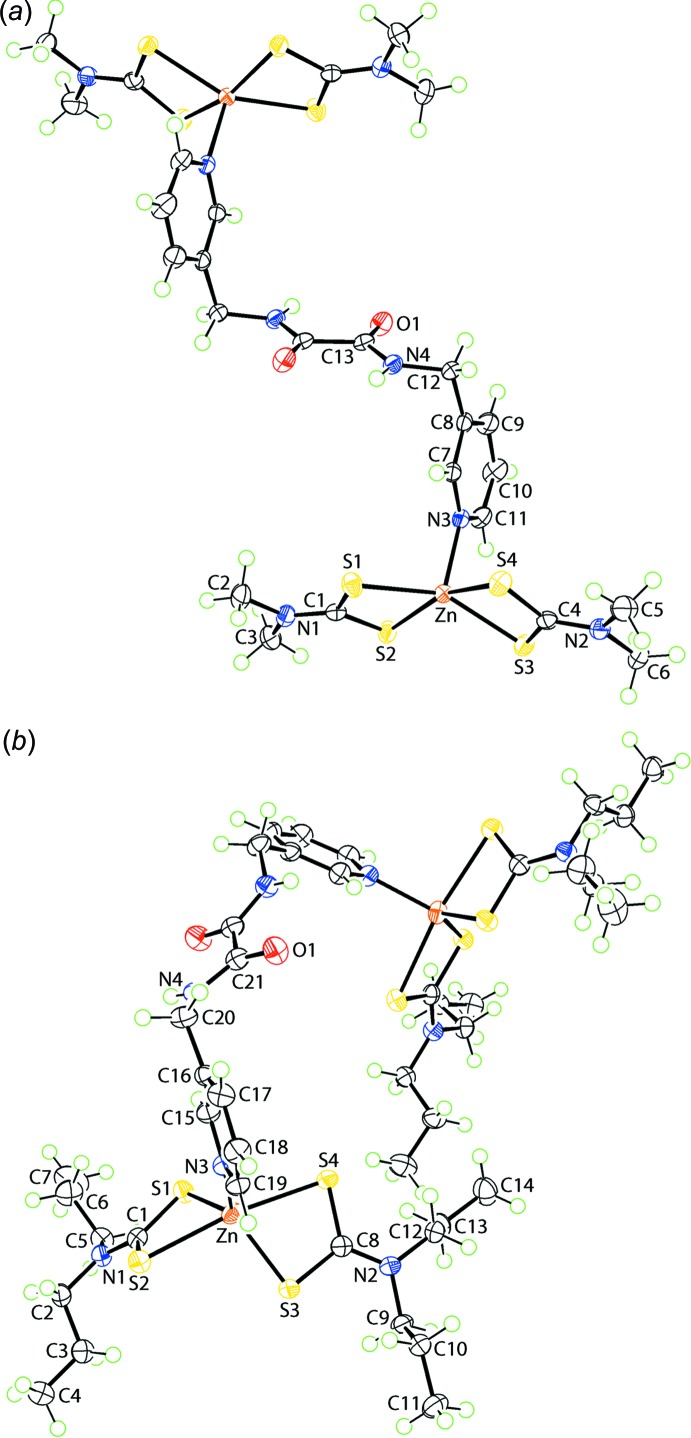
The mol­ecular structures of (*a*) (I) (solvent DMF mol­ecules are omitted) and (*b*) (II) showing the atom-labelling scheme and displacement ellipsoids at the 70% probability level.

The mol­ecular structure of the centrosymmetric, binuclear zinc(II) compound in (I) is shown in Fig. 1 ▸ *a* and selected geometric parameters are collected in Table 1 ▸. The zinc centre is coordinated by two chelating di­thio­carbamate ligands and the coordination geometry is completed by a pyridyl-N atom. The di­thio­carbamate ligands coordinate differently, with the S1-ligand coordinating almost symmetrically with Δ(Zn—S) = (Zn—S_long_ − Zn—S_short_) = 0.10 A. By contrast, the S3-ligand coordinates slightly more asymmetrically with Δ(Zn—S) = 0.18 Å. These differences are not reflected in the associated C—S bond lengths, which span an experimentally equivalent range of 1.720 (2) to 1.732 (2) Å. The resulting NS_4_ donor set defines a distorted square-pyramidal geometry as judged by the value of τ = 0.18 which compares to τ = 0.0 for an ideal square-pyramid and 1.0 for an ideal trigonal-bipyramidal geometry (Addison *et al.*, 1984 ▸). In this description, the zinc atom lies 0.5011 (3) Å above the plane defined by the four sulfur atoms [r.m.s. deviation = 0.0976 Å with the range of deviations being −0.0990 (3) Å for the S3 atom to 0.0987 (3) Å for S2]. The widest angles are defined by the sulfur atoms forming the shorter of the Zn—S bonds of each di­thio­carbamate ligand and by those forming the longer Zn—S bonds. The dihedral angle between the best plane through the four sulfur atoms and that through the pyridyl ring is 87.13 (4)°, indicating a near perpendicular relationship. The dihedral angle between the two chelate rings is 27.46 (6)°.

The mol­ecular structure of the binuclear zinc(II) compound, (II), is shown in Fig. 1 ▸ *b* and again selected geometric parameters are collected in Table 1 ▸. The first and most obvious distinction between the binuclear compounds in (I) and (II) relates to the symmetry within the mol­ecules, *i.e.* the bridging ligand is disposed about a centre of inversion in (I), leading to an extended conformation, but is disposed about a twofold axis in (II), leading to a curved conformation. While to a first approximation the coordination geometry in (II) matches that in (I), some differences are apparent. Each di­thio­carbamate ligand coordinates asymmetrically with Δ(Zn—S) = 0.26 and 0.22 Å, respectively, and these differences are reflected in the associated C—S bond lengths with those associated with the weakly coordinating sulfur atoms being significantly shorter than those associated with the more tightly bound sulfur atoms, Table 1 ▸. There is also a significant difference in the coordination geometry defined by the NS_4_ donor set with τ = 0.76. This difference arises from a reduction, by approximately 25°, of the angle subtended at the zinc atom by the more tightly bound sulfur atoms, Table 1 ▸. The change in coordination geometry is reflected in the relatively wide dihedral angle between the chelate rings of 59.41 (3)°.

The common feature of (I) and (II) is the relatively long central *sp* ^2^-C—C(*sp* ^2^) bond, Table 1 ▸. This feature for these ligands is well established and is reflected by comparable bond lengths determined by experiment and theory for the two polymorphs known for the uncoordinated ligand, ^3^ *L*H_2_ (Jotani, Zukerman-Schpector *et al.*, 2016 ▸). Inter­estingly, in one of the polymorphs, both independent mol­ecules are disposed about a centre of inversion and adopt an *anti*-periplanar form, as in (I), while in the second polymorph, the mol­ecule is twofold symmetric with a U-shaped conformation, *i.e*. is *syn*-periplanar, as in (II). Computational chemistry indicated no significant energy difference between the two conformations, a result consistent with the literature expectation for the majority of conformational polymorphs (Cruz-Cabeza *et al.*, 2015 ▸).

## Supra­molecular features   

**Figure 2:**
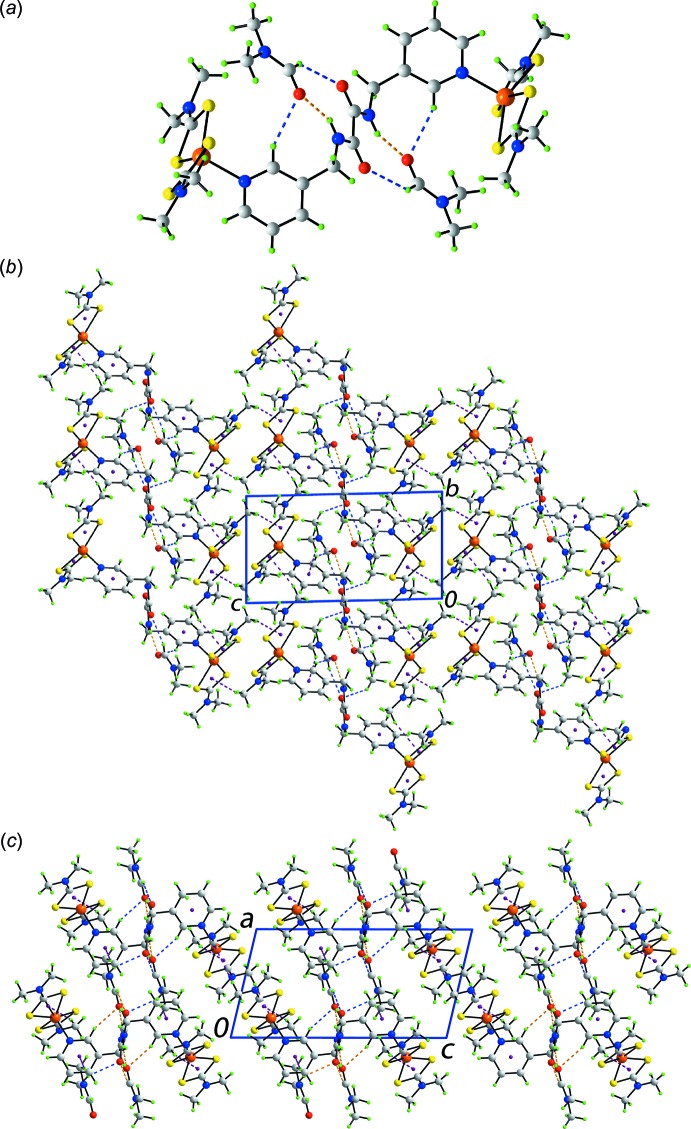
Mol­ecular packing in (I): (*a*) supra­molecular three-mol­ecule aggregate sustained by amide-N—H⋯O(DMF) hydrogen bonding, (*b*) a view of the unit-cell contents in projection down the *a* axis and (*c*) a view of the unit-cell contents in projection down the *b* axis. The N—H⋯O, C—H⋯O and C—H⋯π inter­actions are shown as orange, blue and purple dashed lines, respectively.

The presence of solvent DMF mol­ecules in the crystal of (I) precludes supra­molecular self-association between the amide functionality. Instead, three-mol­ecule aggregates are generated *via* amide-N—H⋯O(DMF) hydrogen bonds, Fig. 2 ▸ *a* and Table 2 ▸. These aggregates are further linked *via* DMF-C—H⋯O(amide) and pyridyl-C—H⋯O(DMF) inter­actions, leading to eight-membered {⋯OC_2_NH⋯OCH} and seven-membered {⋯O⋯HNC_3_H} synthons, respectively. Connections between these aggregates are of the type methyl-C—H⋯π, where the π-systems are either the pyridyl ring or one of the chelate rings. Referring to the latter, such C—H⋯π(chelate) ring inter­actions are more and more being observed in the structural chemistry of metal di­thio­carbamates owing, no doubt, to the effective chelating ability of di­thio­carbamate ligands, which leads to significant π-electron density within the chelate rings they form (Tiekink & Zukerman-Schpector, 2011 ▸; Tiekink, 2017 ▸). The net result of the foregoing is a three-dimensional architecture, Fig. 2 ▸ *b*. From the view down the *b* axis, Fig. 2 ▸ *c*, there are obvious areas with little or no directional inter­actions between the residues.

**Figure 3:**
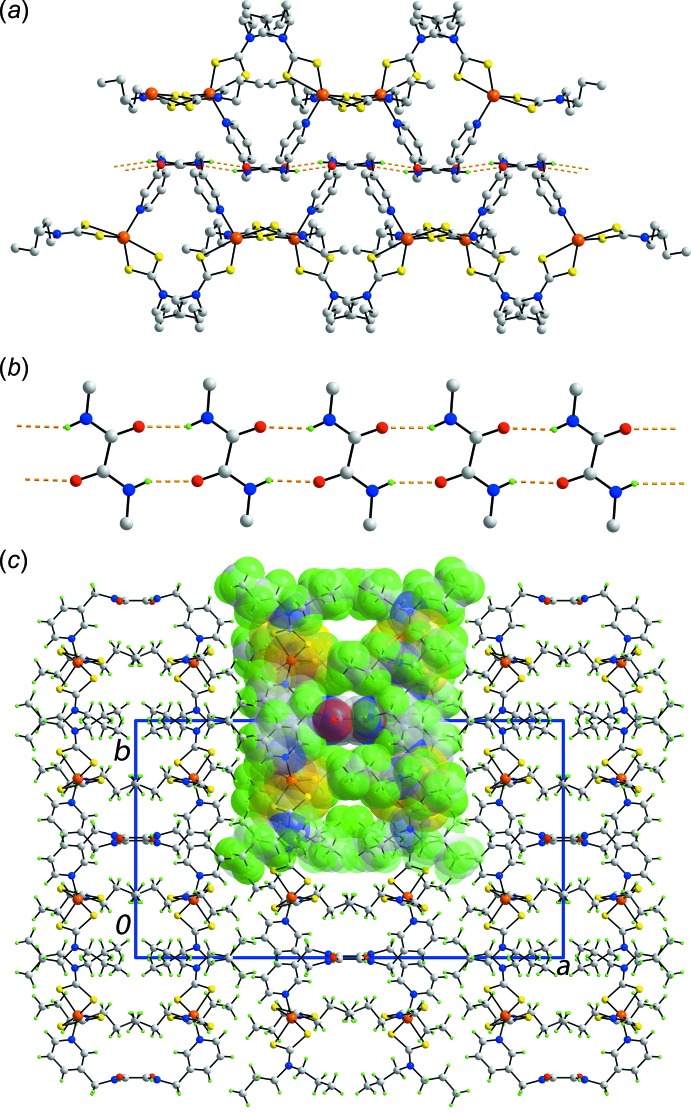
Mol­ecular packing in (II): (*a*) linear supra­molecular chain aligned along the *c* axis and sustained by amide-N—H⋯O(amide) hydrogen bonding (orange dashed lines), with non-participating hydrogen atoms omitted (*b*) detail of the amide-N—H⋯O(amide) hydrogen-bonded tape and (*c*) a view of the unit-cell contents in projection down the *c* axis, with one chain highlighted in space-filling mode.

By contrast to the myriad of supra­molecular associations identified in the crystal of (I), only conventional amide-N—H⋯O(amide) hydrogen bonding is found in the crystal of (II), Table 3 ▸, with no other specific inter­actions identified based on the distance criteria in *PLATON* (Spek, 2009 ▸). The hydrogen bonding leads to linear supra­molecular chains along the *c* axis, Fig. 3 ▸ *a*, with alternate binuclear mol­ecules lying above and below the plane defined by the supra­molecular tape shown in Fig. 3 ▸ *b*. A view of the unit-cell contents, with one chain highlighted in space-filling mode, is shown in Fig. 3 ▸ *c*.

## Database survey   

The investigation of zinc(II) di­thio­carbamates, Zn(S_2_CN*RR*′)_2_, with at least one of *R*/*R*′ being CH_2_CH_2_OH, has lead to an inter­esting array of structures owing to hydrogen bonding. Thus, hy­droxy-O—H⋯O(hy­droxy) hydrogen bonding links otherwise mol­ecular species into supra­molecular chains in the cases of Zn[S_2_CN(*R*)CH_2_CH_2_OH]_2_(pyridine)·pyridine for *R* = Me and Et (Poplaukhin & Tiekink, 2017 ▸) and Zn[S_2_CN(Me)CH_2_CH_2_OH]_2_(3-hy­droxy­pyridine) (Jotani, Arman *et al.*, 2016 ▸) and supra­molecular layers *via* hy­droxy-O—H⋯S(di­thio­carbamate) hydrogen bonds in Zn[S_2_CN(*i*-Pr)CH_2_CH_2_OH]_2_(2,2′-bi­pyridine) (Safbri *et al.*, 2016 ▸); the propensity for the hy­droxy group in di­thio­carbamate ligands with *R* = CH_2_CH_2_OH to form O—H⋯S rather than O—H⋯O hydrogen bonds has been summarized recently (Jamaludin *et al.*, 2016 ▸). With potentially bridging ligands, mixed results have been observed in recent studies: in terms of potentially tetra-coordinate urotropine (hexa­methyl­ene­tetra­amine, hmta), monodentate coordination has been found in each of the four independent mol­ecules comprising the asymmetric unit of Zn[S_2_CN(*i*-Pr)CH_2_CH_2_OH]_2_(hmta) (Câmpian *et al.*, 2016 ▸). Supra­molecular layers are sustained by hy­droxy-O—H⋯O(hy­droxy) and hy­droxy-O—H⋯S(di­thio­carbamate) hydrogen bonding, as per above, augmented by hy­droxy-O—H⋯N(hmta) hydrogen bonding. Bidentate bridging has been found in 2:1 adducts of Zn[S_2_CN(CH_2_CH_2_OH)_2_]_2_}_2_ with pyrazine (Jotani *et al.*, 2017 ▸) and 4,4′-bipyridyl (Benson *et al.*, 2007 ▸) in which three-dimensional architectures are sustained by hy­droxy-O—H⋯O(hy­droxy) hydrogen bonding. Apart from the inter­woven polymers discussed in the *Chemical context*, the most closely related compounds to the title compounds are thio­amide analogues of ^3^ *L*H_2_, *i.e*. ^3^ *L*SH_2_. Some inter­esting crystal chemistry occurs when {Zn[S_2_CN(Me)CH_2_CH_2_OH]_2_}_2_(^3^ *L*SH_2_) is recrystallized from aceto­nitrile (Poplaukhin *et al.*, 2012 ▸). Upon prolonged standing, a one molar ratio of S_8_, a decomposition product, is incorporated in the co-crystal with hy­droxy-O—H⋯O(hy­droxy) hydrogen bonding leading to a two-dimensional array. When DMF is diffused into an aceto­nitrile solution of the same compound, one hy­droxy group hydrogen bonds to the DMF-O while the other hydroxyl group self-associates to form a supra­molecular chain. In the present study, when additional hydrogen-bonding functionality is not present, the amide groups are able to self-assemble as shown in Fig. 3 ▸. With the foregoing in mind, *i.e*. variable coordination geometries, flexible conformations of the bridging ligands and different hydrogen-bonding potential, more systematic studies in this area are warranted.

## Synthesis and crystallization   

Crystals of (I) were grown from liquid diffusion of ether into a 1:1 molar ratio of Zn(S_2_CNMe_2_)_2_ and ^3^ *L*H_2_ in DMF; m.p. 479–481 K. Crystals of (II) were grown from the slow evaporation of a 2:1 molar ratio of Zn[S_2_CN(*n*-Pr)_2_]_2_ and ^3^ *L*H_2_ in a MeOH/EtOH solution.

## Refinement   

Crystal data, data collection and structure refinement details are summarized in Table 4 ▸. For each of (I) and (II), carbon-bound H atoms were placed in calculated positions (C—H = 0.95–0.98 Å) and were included in the refinement in the riding-model approximation, with *U*_iso_(H) set to 1.2–1.5*U*_eq_(C). The N-bound H atoms were located in difference-Fourier maps but were refined with a distance restraint of N—H = 0.88±0.01 Å, and with *U*_iso_(H) set to 1.2*U*_eq_(N). Owing to poor agreement, two reflections, *i.e*. (3
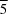
6) and (014), were omitted from the final cycles of refinement of (I).

## Supplementary Material

Crystal structure: contains datablock(s) I, II, global. DOI: 10.1107/S2056989017012956/hb7704sup1.cif

Structure factors: contains datablock(s) I. DOI: 10.1107/S2056989017012956/hb7704Isup2.hkl

Structure factors: contains datablock(s) II. DOI: 10.1107/S2056989017012956/hb7704IIsup3.hkl

CCDC references: 1573823, 1573822

Additional supporting information:  crystallographic information; 3D view; checkCIF report

## Figures and Tables

**Table 1:** Geometric data (Å, °) for (I) and (II)

Parameter	(I); *n* = 4; *m* = 13*^*a*^*	(II); *n* = 8; *m* = 21*^*b*^*
Zn—S1	2.3770 (5)	2.3289 (5)
Zn—S2	2.4784 (5)	2.5917 (6)
Zn—S3	2.3807 (5)	2.3484 (5)
Zn—S4	2.5565 (6)	2.5731 (5)
Zn—N3	2.0959 (16)	2.0595 (15)
C1—S1	1.732 (2)	1.7391 (19)
C1—S2	1.720 (2)	1.715 (2)
C*n*—S3	1.7310 (19)	1.7435 (19)
C*n*—S4	1.7224 (19)	1.7114 (19)
C*m*—O1	1.229 (2)	1.237 (2)
C*m*—N4	1.334 (2)	1.334 (2)
C*m*—C*m* ^i^	1.550 (4)	1.538 (4)
S1—Zn—S3	150.85 (2)	125.23 (2)
S2—Zn—S4	161.48 (2)	170.88 (2)

Notes: (*a*) symmetry code: (i) −*x*, 2 − *y*, 1 − *z*; (*b*) symmetry code: (i) −*x*, *y*, 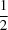 − *z*.

**Table 2:** Hydrogen-bond geometry (Å, °) for (I) *Cg*1–*Cg*3 are the centroids of the Zn/S1/S2/C1, Zn/S3/S4/C4 and N3/C7–C11 rings, respectively.

*D*—H⋯*A*	*D*—H	H⋯*A*	*D*⋯*A*	*D*—H⋯*A*
N4—H4*N*⋯O2	0.87 (2)	1.99 (2)	2.779 (2)	151 (2)
C7—H7⋯O2	0.95	2.44	3.290 (3)	149
C14—H14⋯O1^i^	0.95	2.49	3.245 (2)	137
C15—H15*B*⋯O1^ii^	0.98	2.47	3.310 (3)	144
C3—H3*B*⋯*Cg*2^iii^	0.98	2.93	3.884 (3)	165
C5—H5*B*⋯*Cg*1^iv^	0.98	2.97	3.924 (2)	164
C16—H16*C*⋯*Cg*3^v^	0.98	2.72	3.562 (3)	144

Symmetry codes: (i) 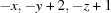; (ii) 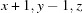; (iii) 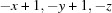; (iv) 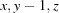; (v) 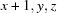.

**Table 3:** Hydrogen-bond geometry (Å, °) for (II)

*D*—H⋯*A*	*D*—H	H⋯*A*	*D*⋯*A*	*D*—H⋯*A*
N4—H4*N*⋯O1^i^	0.87 (2)	2.16 (2)	2.959 (2)	153 (2)

Symmetry code: (i) 
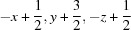
.

**Table 4:** Experimental details

	(I)	(II)
Crystal data		
Chemical formula	[Zn_2_(C_3_H_6_NS_2_)_4_(C_14_H_14_N_4_O_2_)]·2C_3_H_7_NO	[Zn_2_(C_7_H_14_NS_2_)_4_(C_14_H_14_N_4_O_2_)]
*M*_r_	1028.05	1106.28
Crystal system, space group	Triclinic, *P* 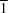	Monoclinic, *C*2/*c*
Temperature (K)	98	98
*a*, *b*, *c* (Å)	9.0998 (8), 9.3544 (10), 15.508 (2)	31.048 (4), 16.923 (2), 10.3453 (14)
α, β, γ (°)	84.176 (1), 75.540 (8), 61.067 (6)	90, 100.361 (2), 90
*V* (Å^3^)	1118.5 (2)	5347.1 (12)
*Z*	1	4
Radiation type	Mo *K*α	Mo *K*α
μ (mm^−1^)	1.49	1.25
Crystal size (mm)	0.22 × 0.16 × 0.07	0.35 × 0.27 × 0.19

Data collection		
Diffractometer	Rigaku AFC12K/SATURN724	Rigaku AFC12K/SATURN724
Absorption correction	Multi-scan (*ABSCOR*; Higashi, 1995 ▸)	Multi-scan (*ABSCOR*; Higashi, 1995 ▸)
*T*_min_, *T*_max_	0.823, 1.000	0.642, 1.000
No. of measured, independent and observed [*I* > 2σ(*I*)] reflections	9634, 5062, 4874	17361, 6129, 5700
*R*_int_	0.026	0.031
(sin θ/λ)_max_ (Å^−1^)	0.650	0.650

Refinement		
*R*[*F* ^2^ > 2σ(*F* ^2^)], *wR*(*F* ^2^), *S*	0.031, 0.084, 1.05	0.033, 0.077, 1.09
No. of reflections	5062	6129
No. of parameters	262	287
No. of restraints	1	1
H-atom treatment	H atoms treated by a mixture of independent and constrained refinement	H atoms treated by a mixture of independent and constrained refinement
Δρ_max_, Δρ_min_ (e Å^−3^)	0.41, −0.51	0.31, −0.39

Computer programs: *CrystalClear* (Molecular Structure Corporation & Rigaku, 2005 ▸), *SHELXS* (Sheldrick, 2008 ▸), *SHELXL2014/7* (Sheldrick, 2015 ▸), *ORTEP-3 for Windows* (Farrugia, 2012 ▸), *DIAMOND* (Brandenburg, 2006 ▸) and *publCIF* (Westrip, 2010 ▸).

## supplementary crystallographic information

### {µ2-N,N'-bis[(pyridin-3-yl)methyl]ethanediamide}tetrakis(dimethylcarbamodithioato)dizinc(II) dimethylformamide disolvate (I) Crystal data

**Table:**

[Zn_2_(C_3_H_6_NS_2_)_4_(C_14_H_14_N_4_O_2_)]·2C_3_H_7_NO	*Z* = 1
*M**_r_* = 1028.05	*F*(000) = 534
Triclinic, *P*1	*D*_x_ = 1.526 Mg m^−^^3^
*a* = 9.0998 (8) Å	Mo *K*α radiation, λ = 0.71073 Å
*b* = 9.3544 (10) Å	Cell parameters from 4952 reflections
*c* = 15.508 (2) Å	θ = 2.6–40.2°
α = 84.176 (1)°	µ = 1.49 mm^−^^1^
β = 75.540 (8)°	*T* = 98 K
γ = 61.067 (6)°	Prism, colourless
*V* = 1118.5 (2) Å^3^	0.22 × 0.16 × 0.07 mm

### {µ2-N,N'-bis[(pyridin-3-yl)methyl]ethanediamide}tetrakis(dimethylcarbamodithioato)dizinc(II) dimethylformamide disolvate (I) Data collection

**Table:**

Rigaku AFC12K/SATURN724 diffractometer	5062 independent reflections
Radiation source: fine-focus sealed tube	4874 reflections with *I* > 2σ(*I*)
Graphite monochromator	*R*_int_ = 0.026
ω scans	θ_max_ = 27.5°, θ_min_ = 2.6°
Absorption correction: multi-scan (ABSCOR; Higashi, 1995)	*h* = −11→10
*T*_min_ = 0.823, *T*_max_ = 1.000	*k* = −12→11
9634 measured reflections	*l* = −20→20

### {µ2-N,N'-bis[(pyridin-3-yl)methyl]ethanediamide}tetrakis(dimethylcarbamodithioato)dizinc(II) dimethylformamide disolvate (I) Refinement

**Table:**

Refinement on *F*^2^	1 restraint
Least-squares matrix: full	H atoms treated by a mixture of independent and constrained refinement
*R*[*F*^2^ > 2σ(*F*^2^)] = 0.031	*w* = 1/[σ^2^(*F*_o_^2^) + (0.0453*P*)^2^ + 0.5471*P*] where *P* = (*F*_o_^2^ + 2*F*_c_^2^)/3
*wR*(*F*^2^) = 0.084	(Δ/σ)_max_ < 0.001
*S* = 1.05	Δρ_max_ = 0.41 e Å^−^^3^
5062 reflections	Δρ_min_ = −0.51 e Å^−^^3^
262 parameters	

### {µ2-N,N'-bis[(pyridin-3-yl)methyl]ethanediamide}tetrakis(dimethylcarbamodithioato)dizinc(II) dimethylformamide disolvate (I) Special details

**Table:**

Geometry. All esds (except the esd in the dihedral angle between two l.s. planes) are estimated using the full covariance matrix. The cell esds are taken into account individually in the estimation of esds in distances, angles and torsion angles; correlations between esds in cell parameters are only used when they are defined by crystal symmetry. An approximate (isotropic) treatment of cell esds is used for estimating esds involving l.s. planes.

### {µ2-N,N'-bis[(pyridin-3-yl)methyl]ethanediamide}tetrakis(dimethylcarbamodithioato)dizinc(II) dimethylformamide disolvate (I) Fractional atomic coordinates and isotropic or equivalent isotropic displacement parameters (Å^2^)

**Table:**

	*x*	*y*	*z*	*U*_iso_*/*U*_eq_	
Zn	0.18051 (3)	0.47082 (2)	0.16514 (2)	0.01527 (7)	
S1	0.41361 (6)	0.51174 (6)	0.17595 (3)	0.02024 (11)	
S2	0.19922 (6)	0.65950 (6)	0.04378 (3)	0.01958 (11)	
S3	0.06877 (6)	0.33365 (6)	0.10167 (3)	0.01858 (11)	
S4	0.23877 (6)	0.20909 (6)	0.25237 (3)	0.02033 (11)	
O1	−0.22504 (17)	1.09018 (16)	0.51306 (10)	0.0212 (3)	
O2	0.28455 (17)	0.53249 (17)	0.44844 (10)	0.0228 (3)	
N1	0.4385 (2)	0.7345 (2)	0.06102 (11)	0.0194 (3)	
N2	0.0654 (2)	0.08356 (19)	0.19845 (11)	0.0183 (3)	
N3	−0.03266 (19)	0.63692 (18)	0.25797 (10)	0.0149 (3)	
N4	−0.0222 (2)	0.82110 (19)	0.49761 (11)	0.0158 (3)	
H4N	0.0877 (14)	0.752 (2)	0.4880 (16)	0.019*	
N5	0.5648 (2)	0.40804 (18)	0.37075 (11)	0.0169 (3)	
C1	0.3596 (2)	0.6454 (2)	0.08945 (12)	0.0170 (3)	
C2	0.5708 (3)	0.7264 (3)	0.10280 (15)	0.0269 (4)	
H2A	0.5225	0.7510	0.1668	0.040*	
H2B	0.6080	0.8064	0.0756	0.040*	
H2C	0.6701	0.6164	0.0938	0.040*	
C3	0.4000 (3)	0.8489 (3)	−0.01199 (14)	0.0243 (4)	
H3A	0.3314	0.8288	−0.0441	0.036*	
H3B	0.5081	0.8333	−0.0529	0.036*	
H3C	0.3347	0.9611	0.0121	0.036*	
C4	0.1196 (2)	0.1949 (2)	0.18628 (12)	0.0159 (3)	
C5	0.1033 (3)	−0.0373 (2)	0.26815 (14)	0.0255 (4)	
H5A	0.1810	−0.0286	0.2986	0.038*	
H5B	0.1587	−0.1471	0.2415	0.038*	
H5C	−0.0042	−0.0174	0.3111	0.038*	
C6	−0.0452 (3)	0.0813 (3)	0.14425 (14)	0.0247 (4)	
H6A	−0.1440	0.1906	0.1452	0.037*	
H6B	−0.0866	0.0031	0.1687	0.037*	
H6C	0.0213	0.0488	0.0828	0.037*	
C7	−0.0245 (2)	0.6452 (2)	0.34223 (12)	0.0145 (3)	
H7	0.0816	0.5761	0.3588	0.017*	
C8	−0.1647 (2)	0.7508 (2)	0.40681 (12)	0.0153 (3)	
C9	−0.3197 (2)	0.8473 (2)	0.38194 (14)	0.0209 (4)	
H9	−0.4194	0.9170	0.4247	0.025*	
C10	−0.3289 (3)	0.8416 (2)	0.29438 (15)	0.0235 (4)	
H10	−0.4336	0.9088	0.2761	0.028*	
C11	−0.1820 (2)	0.7357 (2)	0.23420 (13)	0.0191 (4)	
H11	−0.1873	0.7330	0.1740	0.023*	
C12	−0.1430 (2)	0.7598 (2)	0.49952 (12)	0.0169 (4)	
H12A	−0.2564	0.8328	0.5380	0.020*	
H12B	−0.1002	0.6497	0.5254	0.020*	
C13	−0.0750 (2)	0.9808 (2)	0.50327 (12)	0.0154 (3)	
C14	0.4233 (2)	0.5333 (2)	0.41560 (13)	0.0189 (4)	
H14	0.4289	0.6300	0.4230	0.023*	
C15	0.5633 (3)	0.2589 (2)	0.35369 (14)	0.0220 (4)	
H15A	0.4470	0.2718	0.3775	0.033*	
H15B	0.6450	0.1676	0.3827	0.033*	
H15C	0.5968	0.2367	0.2894	0.033*	
C16	0.7237 (3)	0.4164 (3)	0.33479 (15)	0.0246 (4)	
H16A	0.7471	0.4160	0.2696	0.037*	
H16B	0.8192	0.3218	0.3541	0.037*	
H16C	0.7121	0.5171	0.3564	0.037*	

### {µ2-N,N'-bis[(pyridin-3-yl)methyl]ethanediamide}tetrakis(dimethylcarbamodithioato)dizinc(II) dimethylformamide disolvate (I) Atomic displacement parameters (Å^2^)

**Table:**

	*U*^11^	*U*^22^	*U*^33^	*U*^12^	*U*^13^	*U*^23^
Zn	0.01537 (12)	0.01651 (12)	0.01506 (12)	−0.00876 (9)	−0.00181 (8)	−0.00223 (8)
S1	0.0178 (2)	0.0245 (2)	0.0207 (2)	−0.01150 (19)	−0.00697 (18)	0.00486 (18)
S2	0.0239 (2)	0.0256 (2)	0.0169 (2)	−0.0171 (2)	−0.00714 (18)	0.00306 (17)
S3	0.0260 (2)	0.0194 (2)	0.0159 (2)	−0.01463 (19)	−0.00632 (18)	0.00188 (16)
S4	0.0212 (2)	0.0184 (2)	0.0229 (2)	−0.00868 (18)	−0.00950 (19)	0.00188 (17)
O1	0.0164 (6)	0.0168 (6)	0.0288 (8)	−0.0050 (5)	−0.0074 (6)	−0.0032 (5)
O2	0.0145 (6)	0.0239 (7)	0.0270 (7)	−0.0068 (6)	−0.0030 (6)	−0.0040 (6)
N1	0.0218 (8)	0.0220 (8)	0.0189 (8)	−0.0138 (7)	−0.0051 (6)	0.0002 (6)
N2	0.0227 (8)	0.0163 (7)	0.0169 (8)	−0.0111 (6)	−0.0023 (6)	0.0001 (6)
N3	0.0143 (7)	0.0159 (7)	0.0164 (7)	−0.0086 (6)	−0.0030 (6)	−0.0016 (6)
N4	0.0147 (7)	0.0153 (7)	0.0174 (7)	−0.0065 (6)	−0.0037 (6)	−0.0024 (6)
N5	0.0145 (7)	0.0152 (7)	0.0202 (8)	−0.0065 (6)	−0.0035 (6)	−0.0008 (6)
C1	0.0172 (8)	0.0175 (8)	0.0149 (8)	−0.0079 (7)	−0.0005 (7)	−0.0043 (6)
C2	0.0259 (10)	0.0379 (12)	0.0271 (11)	−0.0219 (10)	−0.0081 (9)	−0.0004 (9)
C3	0.0289 (10)	0.0269 (10)	0.0215 (10)	−0.0188 (9)	−0.0025 (8)	0.0035 (8)
C4	0.0151 (8)	0.0127 (8)	0.0165 (8)	−0.0049 (7)	−0.0005 (7)	−0.0031 (6)
C5	0.0348 (11)	0.0178 (9)	0.0222 (10)	−0.0130 (8)	−0.0038 (9)	0.0033 (7)
C6	0.0321 (11)	0.0264 (10)	0.0240 (10)	−0.0204 (9)	−0.0048 (9)	−0.0030 (8)
C7	0.0144 (8)	0.0148 (8)	0.0160 (8)	−0.0081 (7)	−0.0032 (7)	−0.0001 (6)
C8	0.0155 (8)	0.0141 (8)	0.0191 (9)	−0.0094 (7)	−0.0026 (7)	−0.0016 (6)
C9	0.0155 (9)	0.0170 (8)	0.0267 (10)	−0.0047 (7)	−0.0027 (8)	−0.0061 (7)
C10	0.0181 (9)	0.0189 (9)	0.0306 (11)	−0.0034 (8)	−0.0108 (8)	−0.0036 (8)
C11	0.0199 (9)	0.0194 (8)	0.0205 (9)	−0.0095 (7)	−0.0081 (7)	−0.0011 (7)
C12	0.0183 (8)	0.0168 (8)	0.0170 (9)	−0.0100 (7)	−0.0009 (7)	−0.0043 (6)
C13	0.0176 (9)	0.0164 (8)	0.0125 (8)	−0.0073 (7)	−0.0047 (7)	−0.0022 (6)
C14	0.0175 (9)	0.0171 (8)	0.0195 (9)	−0.0055 (7)	−0.0058 (7)	−0.0001 (7)
C15	0.0220 (9)	0.0174 (9)	0.0261 (10)	−0.0087 (8)	−0.0048 (8)	−0.0032 (7)
C16	0.0199 (9)	0.0260 (10)	0.0274 (10)	−0.0126 (8)	0.0006 (8)	−0.0039 (8)

### {µ2-N,N'-bis[(pyridin-3-yl)methyl]ethanediamide}tetrakis(dimethylcarbamodithioato)dizinc(II) dimethylformamide disolvate (I) Geometric parameters (Å, º)

**Table:**

Zn—N3	2.0959 (16)	C3—H3A	0.9800
Zn—S1	2.3770 (5)	C3—H3B	0.9800
Zn—S3	2.3807 (5)	C3—H3C	0.9800
Zn—S2	2.4784 (5)	C5—H5A	0.9800
Zn—S4	2.5565 (6)	C5—H5B	0.9800
S1—C1	1.732 (2)	C5—H5C	0.9800
S2—C1	1.720 (2)	C6—H6A	0.9800
S3—C4	1.7310 (19)	C6—H6B	0.9800
S4—C4	1.7224 (19)	C6—H6C	0.9800
O1—C13	1.229 (2)	C7—C8	1.396 (2)
O2—C14	1.241 (2)	C7—H7	0.9500
N1—C1	1.324 (2)	C8—C9	1.387 (3)
N1—C3	1.462 (3)	C8—C12	1.515 (3)
N1—C2	1.472 (3)	C9—C10	1.389 (3)
N2—C4	1.332 (2)	C9—H9	0.9500
N2—C5	1.458 (2)	C10—C11	1.386 (3)
N2—C6	1.472 (3)	C10—H10	0.9500
N3—C7	1.339 (2)	C11—H11	0.9500
N3—C11	1.343 (2)	C12—H12A	0.9900
N4—C13	1.334 (2)	C12—H12B	0.9900
N4—C12	1.460 (2)	C13—C13^i^	1.550 (4)
N4—H4N	0.872 (9)	C14—H14	0.9500
N5—C14	1.331 (2)	C15—H15A	0.9800
N5—C15	1.453 (2)	C15—H15B	0.9800
N5—C16	1.451 (2)	C15—H15C	0.9800
C2—H2A	0.9800	C16—H16A	0.9800
C2—H2B	0.9800	C16—H16B	0.9800
C2—H2C	0.9800	C16—H16C	0.9800
			
N3—Zn—S1	104.88 (4)	H5A—C5—H5B	109.5
N3—Zn—S3	104.27 (4)	N2—C5—H5C	109.5
S1—Zn—S3	150.85 (2)	H5A—C5—H5C	109.5
N3—Zn—S2	99.88 (5)	H5B—C5—H5C	109.5
S1—Zn—S2	74.936 (18)	N2—C6—H6A	109.5
S3—Zn—S2	100.297 (19)	N2—C6—H6B	109.5
N3—Zn—S4	98.56 (4)	H6A—C6—H6B	109.5
S1—Zn—S4	101.877 (19)	N2—C6—H6C	109.5
S3—Zn—S4	73.378 (18)	H6A—C6—H6C	109.5
S2—Zn—S4	161.475 (19)	H6B—C6—H6C	109.5
C1—S1—Zn	85.00 (7)	N3—C7—C8	123.02 (17)
C1—S2—Zn	82.11 (7)	N3—C7—H7	118.5
C4—S3—Zn	86.76 (6)	C8—C7—H7	118.5
C4—S4—Zn	81.49 (6)	C9—C8—C7	117.60 (17)
C1—N1—C3	123.25 (17)	C9—C8—C12	122.39 (17)
C1—N1—C2	121.46 (17)	C7—C8—C12	120.00 (16)
C3—N1—C2	115.28 (16)	C8—C9—C10	119.81 (18)
C4—N2—C5	122.90 (17)	C8—C9—H9	120.1
C4—N2—C6	121.21 (16)	C10—C9—H9	120.1
C5—N2—C6	115.82 (16)	C11—C10—C9	118.61 (18)
C7—N3—C11	118.55 (16)	C11—C10—H10	120.7
C7—N3—Zn	121.14 (12)	C9—C10—H10	120.7
C11—N3—Zn	120.29 (13)	N3—C11—C10	122.33 (18)
C13—N4—C12	121.39 (16)	N3—C11—H11	118.8
C13—N4—H4N	119.8 (15)	C10—C11—H11	118.8
C12—N4—H4N	118.6 (15)	N4—C12—C8	111.17 (15)
C14—N5—C15	120.66 (16)	N4—C12—H12A	109.4
C14—N5—C16	121.79 (16)	C8—C12—H12A	109.4
C15—N5—C16	117.52 (16)	N4—C12—H12B	109.4
N1—C1—S2	121.84 (15)	C8—C12—H12B	109.4
N1—C1—S1	120.43 (15)	H12A—C12—H12B	108.0
S2—C1—S1	117.73 (11)	O1—C13—N4	125.76 (17)
N1—C2—H2A	109.5	O1—C13—C13^i^	121.4 (2)
N1—C2—H2B	109.5	N4—C13—C13^i^	112.88 (19)
H2A—C2—H2B	109.5	O2—C14—N5	124.57 (18)
N1—C2—H2C	109.5	O2—C14—H14	117.7
H2A—C2—H2C	109.5	N5—C14—H14	117.7
H2B—C2—H2C	109.5	N5—C15—H15A	109.5
N1—C3—H3A	109.5	N5—C15—H15B	109.5
N1—C3—H3B	109.5	H15A—C15—H15B	109.5
H3A—C3—H3B	109.5	N5—C15—H15C	109.5
N1—C3—H3C	109.5	H15A—C15—H15C	109.5
H3A—C3—H3C	109.5	H15B—C15—H15C	109.5
H3B—C3—H3C	109.5	N5—C16—H16A	109.5
N2—C4—S4	122.67 (15)	N5—C16—H16B	109.5
N2—C4—S3	119.78 (15)	H16A—C16—H16B	109.5
S4—C4—S3	117.56 (10)	N5—C16—H16C	109.5
N2—C5—H5A	109.5	H16A—C16—H16C	109.5
N2—C5—H5B	109.5	H16B—C16—H16C	109.5
			
C3—N1—C1—S2	−1.3 (3)	Zn—N3—C7—C8	−178.18 (13)
C2—N1—C1—S2	177.58 (15)	N3—C7—C8—C9	1.8 (3)
C3—N1—C1—S1	179.60 (14)	N3—C7—C8—C12	−176.74 (16)
C2—N1—C1—S1	−1.5 (3)	C7—C8—C9—C10	−2.8 (3)
Zn—S2—C1—N1	−174.64 (16)	C12—C8—C9—C10	175.73 (18)
Zn—S2—C1—S1	4.45 (9)	C8—C9—C10—C11	1.4 (3)
Zn—S1—C1—N1	174.49 (16)	C7—N3—C11—C10	−2.2 (3)
Zn—S1—C1—S2	−4.61 (10)	Zn—N3—C11—C10	176.66 (15)
C5—N2—C4—S4	−0.6 (3)	C9—C10—C11—N3	1.2 (3)
C6—N2—C4—S4	176.20 (14)	C13—N4—C12—C8	88.2 (2)
C5—N2—C4—S3	179.90 (14)	C9—C8—C12—N4	−114.59 (19)
C6—N2—C4—S3	−3.3 (2)	C7—C8—C12—N4	63.9 (2)
Zn—S4—C4—N2	−171.25 (16)	C12—N4—C13—O1	2.3 (3)
Zn—S4—C4—S3	8.27 (9)	C12—N4—C13—C13^i^	−178.06 (18)
Zn—S3—C4—N2	170.73 (15)	C15—N5—C14—O2	2.6 (3)
Zn—S3—C4—S4	−8.80 (10)	C16—N5—C14—O2	−179.34 (19)
C11—N3—C7—C8	0.6 (3)		

Symmetry code: (i) −*x*, −*y*+2, −*z*+1.

### {µ2-N,N'-bis[(pyridin-3-yl)methyl]ethanediamide}tetrakis(dimethylcarbamodithioato)dizinc(II) dimethylformamide disolvate (I) Hydrogen-bond geometry (Å, º)

Cg1–Cg3 are the centroids of the Zn/S1/S2/C1, Zn/S3/S4/C4 and N3/C7–C11 rings, respectively.

**Table:**

*D*—H···*A*	*D*—H	H···*A*	*D*···*A*	*D*—H···*A*
N4—H4*N*···O2	0.87 (2)	1.99 (2)	2.779 (2)	151 (2)
C7—H7···O2	0.95	2.44	3.290 (3)	149
C14—H14···O1^i^	0.95	2.49	3.245 (2)	137
C15—H15*B*···O1^ii^	0.98	2.47	3.310 (3)	144
C3—H3*B*···*Cg*2^iii^	0.98	2.93	3.884 (3)	165
C5—H5*B*···*Cg*1^iv^	0.98	2.97	3.924 (2)	164
C16—H16*C*···*Cg*3^v^	0.98	2.72	3.562 (3)	144

Symmetry codes: (i) −*x*, −*y*+2, −*z*+1; (ii) *x*+1, *y*−1, *z*; (iii) −*x*+1, −*y*+1, −*z*; (iv) *x*, *y*−1, *z*; (v) *x*+1, *y*, *z*.

### {µ2-N,N'-Bis[(pyridin-3-yl)methyl]ethanediamide}tetrakis(di-n-propylcarbamodithioato)dizinc(II) (II) Crystal data

**Table:**

[Zn_2_(C_7_H_14_NS_2_)_4_(C_14_H_14_N_4_O_2_)]	*F*(000) = 2328
*M**_r_* = 1106.28	*D*_x_ = 1.374 Mg m^−^^3^
Monoclinic, *C*2/*c*	Mo *K*α radiation, λ = 0.71073 Å
*a* = 31.048 (4) Å	Cell parameters from 12374 reflections
*b* = 16.923 (2) Å	θ = 2.3–40.8°
*c* = 10.3453 (14) Å	µ = 1.25 mm^−^^1^
β = 100.361 (2)°	*T* = 98 K
*V* = 5347.1 (12) Å^3^	Prism, colourless
*Z* = 4	0.35 × 0.27 × 0.19 mm

### {µ2-N,N'-Bis[(pyridin-3-yl)methyl]ethanediamide}tetrakis(di-n-propylcarbamodithioato)dizinc(II) (II) Data collection

**Table:**

Rigaku AFC12K/SATURN724 diffractometer	6129 independent reflections
Radiation source: fine-focus sealed tube	5700 reflections with *I* > 2σ(*I*)
Graphite monochromator	*R*_int_ = 0.031
ω scans	θ_max_ = 27.5°, θ_min_ = 2.3°
Absorption correction: multi-scan (ABSCOR; Higashi, 1995)	*h* = −40→30
*T*_min_ = 0.642, *T*_max_ = 1.000	*k* = −21→21
17361 measured reflections	*l* = −13→13

### {µ2-N,N'-Bis[(pyridin-3-yl)methyl]ethanediamide}tetrakis(di-n-propylcarbamodithioato)dizinc(II) (II) Refinement

**Table:**

Refinement on *F*^2^	1 restraint
Least-squares matrix: full	H atoms treated by a mixture of independent and constrained refinement
*R*[*F*^2^ > 2σ(*F*^2^)] = 0.033	*w* = 1/[σ^2^(*F*_o_^2^) + (0.0308*P*)^2^ + 4.5145*P*] where *P* = (*F*_o_^2^ + 2*F*_c_^2^)/3
*wR*(*F*^2^) = 0.077	(Δ/σ)_max_ = 0.001
*S* = 1.09	Δρ_max_ = 0.31 e Å^−^^3^
6129 reflections	Δρ_min_ = −0.39 e Å^−^^3^
287 parameters	

### {µ2-N,N'-Bis[(pyridin-3-yl)methyl]ethanediamide}tetrakis(di-n-propylcarbamodithioato)dizinc(II) (II) Special details

**Table:**

Geometry. All esds (except the esd in the dihedral angle between two l.s. planes) are estimated using the full covariance matrix. The cell esds are taken into account individually in the estimation of esds in distances, angles and torsion angles; correlations between esds in cell parameters are only used when they are defined by crystal symmetry. An approximate (isotropic) treatment of cell esds is used for estimating esds involving l.s. planes.

### {µ2-N,N'-Bis[(pyridin-3-yl)methyl]ethanediamide}tetrakis(di-n-propylcarbamodithioato)dizinc(II) (II) Fractional atomic coordinates and isotropic or equivalent isotropic displacement parameters (Å^2^)

**Table:**

	*x*	*y*	*z*	*U*_iso_*/*U*_eq_	
Zn	0.13541 (2)	0.24490 (2)	0.60218 (2)	0.01873 (7)	
S1	0.08181 (2)	0.25161 (3)	0.73301 (4)	0.01949 (10)	
S2	0.17501 (2)	0.28982 (3)	0.83257 (4)	0.01932 (10)	
S3	0.17753 (2)	0.13268 (2)	0.57617 (4)	0.01802 (10)	
S4	0.09814 (2)	0.17783 (3)	0.38786 (4)	0.01878 (10)	
O1	0.03808 (5)	0.50349 (8)	0.14247 (13)	0.0254 (3)	
N1	0.11727 (5)	0.25613 (8)	0.98790 (15)	0.0180 (3)	
N2	0.14225 (5)	0.04388 (9)	0.36979 (14)	0.0178 (3)	
N3	0.14982 (5)	0.34572 (9)	0.50690 (14)	0.0181 (3)	
N4	0.04959 (5)	0.51477 (9)	0.36629 (15)	0.0196 (3)	
H4N	0.0375 (7)	0.5160 (12)	0.4359 (15)	0.024*	
C1	0.12447 (6)	0.26513 (10)	0.86517 (17)	0.0178 (3)	
C2	0.15269 (6)	0.26758 (11)	1.10193 (17)	0.0199 (4)	
H2A	0.1397	0.2798	1.1804	0.024*	
H2B	0.1708	0.3133	1.0852	0.024*	
C3	0.18182 (7)	0.19481 (11)	1.12973 (17)	0.0215 (4)	
H3A	0.1636	0.1490	1.1458	0.026*	
H3B	0.1949	0.1828	1.0514	0.026*	
C4	0.21819 (7)	0.20604 (12)	1.24757 (18)	0.0243 (4)	
H4A	0.2338	0.2554	1.2378	0.036*	
H4B	0.2387	0.1616	1.2532	0.036*	
H4C	0.2056	0.2083	1.3278	0.036*	
C5	0.07487 (6)	0.22987 (11)	1.01677 (18)	0.0211 (4)	
H5A	0.0800	0.1975	1.0978	0.025*	
H5B	0.0603	0.1959	0.9440	0.025*	
C6	0.04457 (7)	0.29765 (13)	1.0347 (2)	0.0285 (4)	
H6A	0.0384	0.3295	0.9532	0.034*	
H6B	0.0589	0.3323	1.1068	0.034*	
C7	0.00182 (8)	0.26606 (17)	1.0668 (2)	0.0407 (6)	
H7A	−0.0130	0.2338	0.9935	0.061*	
H7B	−0.0171	0.3104	1.0813	0.061*	
H7C	0.0080	0.2336	1.1465	0.061*	
C8	0.13929 (6)	0.11097 (10)	0.43552 (16)	0.0169 (3)	
C9	0.17538 (6)	−0.01696 (10)	0.41526 (17)	0.0192 (4)	
H9A	0.1633	−0.0694	0.3855	0.023*	
H9B	0.1816	−0.0172	0.5125	0.023*	
C10	0.21794 (6)	−0.00422 (11)	0.36546 (17)	0.0205 (4)	
H10A	0.2313	0.0464	0.4000	0.025*	
H10B	0.2119	−0.0011	0.2683	0.025*	
C11	0.24983 (7)	−0.07212 (12)	0.4094 (2)	0.0274 (4)	
H11A	0.2547	−0.0766	0.5053	0.041*	
H11B	0.2777	−0.0616	0.3809	0.041*	
H11C	0.2374	−0.1216	0.3699	0.041*	
C12	0.11177 (6)	0.02471 (10)	0.24797 (17)	0.0193 (4)	
H12A	0.1276	−0.0055	0.1893	0.023*	
H12B	0.1013	0.0745	0.2026	0.023*	
C13	0.07247 (7)	−0.02322 (12)	0.27126 (18)	0.0242 (4)	
H13A	0.0561	0.0068	0.3289	0.029*	
H13B	0.0826	−0.0733	0.3160	0.029*	
C14	0.04240 (7)	−0.04110 (13)	0.1408 (2)	0.0309 (5)	
H14A	0.0313	0.0085	0.0987	0.046*	
H14B	0.0178	−0.0736	0.1568	0.046*	
H14C	0.0588	−0.0697	0.0831	0.046*	
C15	0.12006 (6)	0.40358 (10)	0.47673 (17)	0.0192 (4)	
H15	0.0928	0.3991	0.5059	0.023*	
C16	0.12765 (6)	0.46989 (10)	0.40430 (17)	0.0180 (3)	
C17	0.16739 (7)	0.47512 (11)	0.36094 (18)	0.0220 (4)	
H17	0.1734	0.5191	0.3099	0.026*	
C18	0.19815 (7)	0.41597 (11)	0.39255 (18)	0.0232 (4)	
H18	0.2255	0.4188	0.3640	0.028*	
C19	0.18838 (6)	0.35269 (11)	0.46629 (18)	0.0211 (4)	
H19	0.2097	0.3125	0.4892	0.025*	
C20	0.09527 (6)	0.53720 (11)	0.38052 (19)	0.0218 (4)	
H20A	0.1021	0.5748	0.4546	0.026*	
H20B	0.0995	0.5654	0.2999	0.026*	
C21	0.02463 (6)	0.50634 (10)	0.24787 (18)	0.0195 (4)	

### {µ2-N,N'-Bis[(pyridin-3-yl)methyl]ethanediamide}tetrakis(di-n-propylcarbamodithioato)dizinc(II) (II) Atomic displacement parameters (Å^2^)

**Table:**

	*U*^11^	*U*^22^	*U*^33^	*U*^12^	*U*^13^	*U*^23^
Zn	0.02188 (13)	0.01688 (11)	0.01907 (11)	0.00339 (8)	0.00807 (9)	0.00169 (7)
S1	0.0158 (2)	0.0254 (2)	0.0174 (2)	−0.00087 (17)	0.00319 (17)	−0.00181 (16)
S2	0.0158 (2)	0.0231 (2)	0.0198 (2)	−0.00156 (17)	0.00530 (17)	−0.00061 (16)
S3	0.0171 (2)	0.0186 (2)	0.01796 (19)	0.00329 (16)	0.00209 (16)	0.00007 (15)
S4	0.0176 (2)	0.0201 (2)	0.0186 (2)	0.00508 (17)	0.00326 (17)	0.00033 (16)
O1	0.0185 (7)	0.0347 (7)	0.0239 (6)	−0.0012 (6)	0.0066 (6)	−0.0023 (6)
N1	0.0154 (8)	0.0209 (7)	0.0178 (7)	−0.0012 (6)	0.0038 (6)	−0.0018 (5)
N2	0.0154 (8)	0.0185 (7)	0.0193 (7)	0.0030 (6)	0.0026 (6)	0.0001 (6)
N3	0.0170 (8)	0.0176 (7)	0.0201 (7)	0.0010 (6)	0.0039 (6)	0.0007 (6)
N4	0.0142 (8)	0.0216 (7)	0.0227 (7)	0.0008 (6)	0.0021 (6)	0.0011 (6)
C1	0.0183 (9)	0.0166 (8)	0.0191 (8)	0.0009 (7)	0.0048 (7)	−0.0009 (6)
C2	0.0201 (10)	0.0221 (8)	0.0175 (8)	−0.0009 (7)	0.0037 (7)	−0.0032 (7)
C3	0.0218 (10)	0.0227 (9)	0.0208 (8)	−0.0008 (7)	0.0059 (7)	0.0006 (7)
C4	0.0210 (10)	0.0285 (10)	0.0234 (9)	−0.0005 (8)	0.0039 (8)	0.0043 (7)
C5	0.0190 (10)	0.0250 (9)	0.0200 (8)	−0.0034 (7)	0.0054 (7)	0.0014 (7)
C6	0.0201 (11)	0.0343 (11)	0.0333 (10)	0.0011 (8)	0.0105 (9)	0.0002 (8)
C7	0.0202 (12)	0.0690 (16)	0.0351 (12)	0.0017 (11)	0.0112 (9)	0.0086 (11)
C8	0.0159 (9)	0.0190 (8)	0.0172 (8)	−0.0003 (7)	0.0063 (7)	0.0022 (6)
C9	0.0196 (10)	0.0161 (8)	0.0219 (8)	0.0051 (7)	0.0039 (7)	0.0013 (6)
C10	0.0199 (10)	0.0213 (8)	0.0201 (8)	0.0028 (7)	0.0034 (7)	−0.0008 (7)
C11	0.0243 (11)	0.0255 (10)	0.0325 (10)	0.0068 (8)	0.0052 (8)	−0.0031 (8)
C12	0.0201 (10)	0.0202 (8)	0.0181 (8)	0.0016 (7)	0.0048 (7)	−0.0014 (6)
C13	0.0210 (10)	0.0279 (10)	0.0239 (9)	−0.0040 (8)	0.0048 (8)	−0.0038 (7)
C14	0.0237 (11)	0.0393 (11)	0.0287 (10)	−0.0025 (9)	0.0027 (9)	−0.0070 (9)
C15	0.0160 (9)	0.0200 (8)	0.0222 (8)	0.0008 (7)	0.0045 (7)	0.0009 (7)
C16	0.0148 (9)	0.0173 (8)	0.0207 (8)	−0.0004 (7)	0.0000 (7)	−0.0004 (6)
C17	0.0191 (10)	0.0222 (9)	0.0246 (9)	−0.0029 (7)	0.0034 (7)	0.0038 (7)
C18	0.0158 (10)	0.0282 (9)	0.0268 (9)	0.0002 (8)	0.0071 (8)	−0.0001 (7)
C19	0.0179 (10)	0.0219 (9)	0.0234 (9)	0.0037 (7)	0.0036 (7)	−0.0005 (7)
C20	0.0146 (9)	0.0187 (8)	0.0312 (9)	−0.0013 (7)	0.0013 (7)	0.0029 (7)
C21	0.0170 (10)	0.0170 (8)	0.0245 (9)	−0.0008 (7)	0.0035 (7)	−0.0002 (6)

### {µ2-N,N'-Bis[(pyridin-3-yl)methyl]ethanediamide}tetrakis(di-n-propylcarbamodithioato)dizinc(II) (II) Geometric parameters (Å, º)

**Table:**

Zn—N3	2.0595 (15)	C6—H6B	0.9900
Zn—S1	2.3289 (5)	C7—H7A	0.9800
Zn—S3	2.3484 (5)	C7—H7B	0.9800
Zn—S4	2.5731 (5)	C7—H7C	0.9800
Zn—S2	2.5917 (6)	C9—C10	1.518 (3)
S1—C1	1.7391 (19)	C9—H9A	0.9900
S2—C1	1.715 (2)	C9—H9B	0.9900
S3—C8	1.7435 (19)	C10—C11	1.531 (3)
S4—C8	1.7114 (19)	C10—H10A	0.9900
O1—C21	1.237 (2)	C10—H10B	0.9900
N1—C1	1.337 (2)	C11—H11A	0.9800
N1—C5	1.470 (2)	C11—H11B	0.9800
N1—C2	1.474 (2)	C11—H11C	0.9800
N2—C8	1.335 (2)	C12—C13	1.520 (3)
N2—C12	1.470 (2)	C12—H12A	0.9900
N2—C9	1.472 (2)	C12—H12B	0.9900
N3—C19	1.343 (2)	C13—C14	1.527 (3)
N3—C15	1.344 (2)	C13—H13A	0.9900
N4—C21	1.334 (2)	C13—H13B	0.9900
N4—C20	1.450 (2)	C14—H14A	0.9800
N4—H4N	0.870 (9)	C14—H14B	0.9800
C2—C3	1.524 (3)	C14—H14C	0.9800
C2—H2A	0.9900	C15—C16	1.393 (2)
C2—H2B	0.9900	C15—H15	0.9500
C3—C4	1.517 (3)	C16—C17	1.389 (3)
C3—H3A	0.9900	C16—C20	1.510 (3)
C3—H3B	0.9900	C17—C18	1.382 (3)
C4—H4A	0.9800	C17—H17	0.9500
C4—H4B	0.9800	C18—C19	1.380 (3)
C4—H4C	0.9800	C18—H18	0.9500
C5—C6	1.516 (3)	C19—H19	0.9500
C5—H5A	0.9900	C20—H20A	0.9900
C5—H5B	0.9900	C20—H20B	0.9900
C6—C7	1.522 (3)	C21—C21^i^	1.538 (4)
C6—H6A	0.9900		
			
N3—Zn—S1	118.51 (4)	H7B—C7—H7C	109.5
N3—Zn—S3	116.21 (5)	N2—C8—S4	122.11 (14)
S1—Zn—S3	125.225 (18)	N2—C8—S3	120.36 (14)
N3—Zn—S4	93.20 (4)	S4—C8—S3	117.52 (10)
S1—Zn—S4	105.351 (19)	N2—C9—C10	113.16 (14)
S3—Zn—S4	73.615 (17)	N2—C9—H9A	108.9
N3—Zn—S2	95.11 (4)	C10—C9—H9A	108.9
S1—Zn—S2	73.841 (18)	N2—C9—H9B	108.9
S3—Zn—S2	99.247 (17)	C10—C9—H9B	108.9
S4—Zn—S2	170.877 (16)	H9A—C9—H9B	107.8
C1—S1—Zn	86.60 (6)	C9—C10—C11	110.41 (15)
C1—S2—Zn	79.03 (6)	C9—C10—H10A	109.6
C8—S3—Zn	87.51 (6)	C11—C10—H10A	109.6
C8—S4—Zn	81.18 (6)	C9—C10—H10B	109.6
C1—N1—C5	122.43 (16)	C11—C10—H10B	109.6
C1—N1—C2	121.12 (16)	H10A—C10—H10B	108.1
C5—N1—C2	116.34 (14)	C10—C11—H11A	109.5
C8—N2—C12	122.13 (15)	C10—C11—H11B	109.5
C8—N2—C9	122.71 (15)	H11A—C11—H11B	109.5
C12—N2—C9	115.15 (14)	C10—C11—H11C	109.5
C19—N3—C15	118.52 (16)	H11A—C11—H11C	109.5
C19—N3—Zn	120.34 (12)	H11B—C11—H11C	109.5
C15—N3—Zn	121.01 (12)	N2—C12—C13	113.16 (14)
C21—N4—C20	121.14 (16)	N2—C12—H12A	108.9
C21—N4—H4N	119.8 (16)	C13—C12—H12A	108.9
C20—N4—H4N	118.0 (15)	N2—C12—H12B	108.9
N1—C1—S2	121.99 (15)	C13—C12—H12B	108.9
N1—C1—S1	119.86 (15)	H12A—C12—H12B	107.8
S2—C1—S1	118.16 (10)	C12—C13—C14	110.26 (16)
N1—C2—C3	112.14 (14)	C12—C13—H13A	109.6
N1—C2—H2A	109.2	C14—C13—H13A	109.6
C3—C2—H2A	109.2	C12—C13—H13B	109.6
N1—C2—H2B	109.2	C14—C13—H13B	109.6
C3—C2—H2B	109.2	H13A—C13—H13B	108.1
H2A—C2—H2B	107.9	C13—C14—H14A	109.5
C4—C3—C2	112.39 (15)	C13—C14—H14B	109.5
C4—C3—H3A	109.1	H14A—C14—H14B	109.5
C2—C3—H3A	109.1	C13—C14—H14C	109.5
C4—C3—H3B	109.1	H14A—C14—H14C	109.5
C2—C3—H3B	109.1	H14B—C14—H14C	109.5
H3A—C3—H3B	107.9	N3—C15—C16	122.43 (17)
C3—C4—H4A	109.5	N3—C15—H15	118.8
C3—C4—H4B	109.5	C16—C15—H15	118.8
H4A—C4—H4B	109.5	C17—C16—C15	118.11 (17)
C3—C4—H4C	109.5	C17—C16—C20	120.22 (16)
H4A—C4—H4C	109.5	C15—C16—C20	121.55 (17)
H4B—C4—H4C	109.5	C18—C17—C16	119.57 (17)
N1—C5—C6	113.23 (16)	C18—C17—H17	120.2
N1—C5—H5A	108.9	C16—C17—H17	120.2
C6—C5—H5A	108.9	C19—C18—C17	118.81 (18)
N1—C5—H5B	108.9	C19—C18—H18	120.6
C6—C5—H5B	108.9	C17—C18—H18	120.6
H5A—C5—H5B	107.7	N3—C19—C18	122.55 (17)
C5—C6—C7	110.23 (18)	N3—C19—H19	118.7
C5—C6—H6A	109.6	C18—C19—H19	118.7
C7—C6—H6A	109.6	N4—C20—C16	115.44 (15)
C5—C6—H6B	109.6	N4—C20—H20A	108.4
C7—C6—H6B	109.6	C16—C20—H20A	108.4
H6A—C6—H6B	108.1	N4—C20—H20B	108.4
C6—C7—H7A	109.5	C16—C20—H20B	108.4
C6—C7—H7B	109.5	H20A—C20—H20B	107.5
H7A—C7—H7B	109.5	O1—C21—N4	125.54 (18)
C6—C7—H7C	109.5	O1—C21—C21^i^	121.4 (2)
H7A—C7—H7C	109.5	N4—C21—C21^i^	112.96 (19)

Symmetry code: (i) −*x*, *y*, −*z*+1/2.

### {µ2-N,N'-Bis[(pyridin-3-yl)methyl]ethanediamide}tetrakis(di-n-propylcarbamodithioato)dizinc(II) (II) Hydrogen-bond geometry (Å, º)

**Table:**

*D*—H···*A*	*D*—H	H···*A*	*D*···*A*	*D*—H···*A*
N4—H4*N*···O1^ii^	0.87 (2)	2.16 (2)	2.959 (2)	153 (2)

Symmetry code: (ii) −*x*+1/2, *y*+3/2, −*z*+1/2.
